# The Walking Egg non-profit organization

**Published:** 2019-06

**Authors:** W Ombelet, J Onofre, R Campo

**Affiliations:** Chairman of The Walking Egg npo;; Genk Institute for Fertility Technology, Department of Obstetrics and Gynaecology, ZOL Hospitals, Genk, Belgium;; Faculty of Medicine and Life Sciences, Hasselt University, Hasselt, Belgium;; Life Expert Centre, Leuven, Belgium

**Keywords:** Assisted reproduction, low-cost IVF, accessible, affordable, childlessness, developing countries, infertility care, resource-poor countries

## Abstract

The Walking Egg non-profit organization (npo) was founded in 2010 by scientists and an artist. From this unusual blend, a unique objective came forth: to implement accessible infertility programmes in resource-poor countries. Our project aims to raise universal awareness about the issue of childlessness and to make, in all its aspects, infertility care including assisted reproductive technologies (ART), available and accessible worldwide. This can only be achieved when good quality but affordable infertility care is associated with effective family planning and safe motherhood programmes. To be successful, a global project to tackle the socio-cultural, ethical, economical and political differences hindering access to fertility care must happen.

## Introduction

Infertility is a recognized global reproductive health problem. Worldwide a silent population of more than 180 million couples is facing the consequences of childlessness day by day (Rutstein and Iqbal, 2004; Boivin et al., 2007). The consequences of involuntary childlessness are usually more dramatic in developing countries (DC) when compared to Western societies, particularly for women. Indeed, considering the magnitude of childlessness, striking differences between the developed and developing world exist. These can be explained by differences in (a) socio-cultural values surrounding procreation, (in)fertility, and childlessness (b) the economical consequences of being childless and (c) the availability and affordability of infertility treatments.

In several resource-poor countries, about 10 % of all visits to doctors are related to problems of childlessness (Bergstrom, 1992). The problem is mostly underestimated or neglected by health care providers because of lack of knowledge and training in fertility issues, but also due to the fact that assisted reproduction is always associated with expensive techniques and medication, not mentioning the complications that these treatments may engender (e.g. ovarian hyperstimulation syndrome, high-order multiple pregnancies, ..).

Yet, individual health needs of impoverished people do have an echo in public health systems. Although reproductive health education and prevention of infertility are number one priorities in many societies, accessible diagnostic procedures and new simplified reproductive technologies remain a dire need. The success and sustainability of these technologies in resource-poor settings will depend, to a large extend, on our ability to optimise these techniques in terms of safety, availability, affordability and effectiveness.

## History

**Figure 1:**
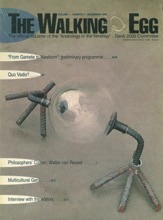
First issue of the international magazine ‘The Walking Egg’, published in March 2000.

The first steps of the Walking Egg date back from 1997 when the cooperation between the scientist Willem Ombelet and artist Koen Vanmechelen resulted in an enigmatic glass egg with the legs of a chicken. This versatile idea first conceived an international magazine (Figure 1) with this same name and 13 years later, the Walking Egg non-profit organization (TWE npo) came to light.

TWE npo was founded in 2010 and right from its beginning opted for a multidisciplinary approach towards the problem of infertility on a global scale. In a unique cooperation with the Special Task Force (STF) on “DC and infertility” of the European Society of Human Reproduction and Embryology (ESHRE) and the WHO (World Health Organization) the main objective of The Walking Egg project was defined: to raise awareness surrounding childlessness, especially in resource-poor countries and to make infertility care in all its aspects, including ART, available and affordable for a much larger part of the world population. This objective could only succeed if the calculus of infertility care in terms of availability, affordability and effectiveness is radically changed from how it is currently provided (Ombelet and Campo, 2007).

## Arusha: the kick-off meeting

**Figure 2:**
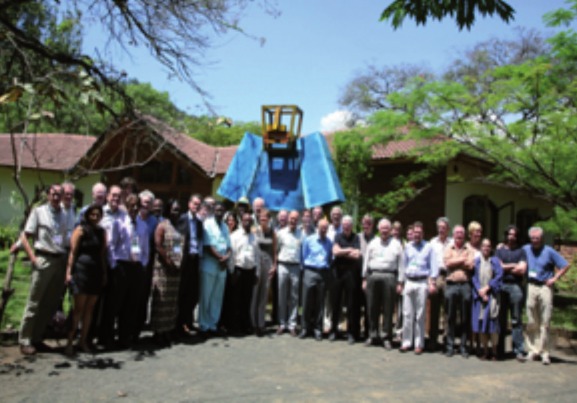
December 2007 - Arusha meeting with 37 experts from different disciplines and 21 different countries.

**Figure 3:**
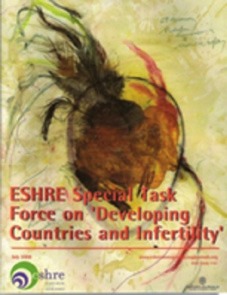
Cover of the ESHRE Monograph pubished in 2008 (designed by Koen Vanmechelen).

In December 2007, a scientific-artistic project in cooperation with the ESHRE STF and the WHO gathered 37 experts in Arusha (Tanzania) to discuss the problem of childlessness and infertility care in resource-poor countries (Figure 2). The content of this meeting was published in a special Monograph of Human Reproduction (Figure 3, Ombelet et al., ESHRE Monograph, 2008). Throughout two days of interactive and captivating discussions it was concluded that for achieving infertility care at a global scale, a substantial number of crucial actions and objectives, in the lines of reproductive health and family planning needed to be addressed (Table I).

**Table I:** — Universal access to infertility care: actions.

To study the ethical and sociocultural aspects of childlessness on a global scale
Investigating the economical aspects of childlessness in developing countries
Measures to promote and support Reproductive Health Care Education, including family planning and mother care
Simplifying techniques of diagnosis of infertility: a simplified “one-stop clinic”
Developing low-cost and effective ovarian stimulation protocols for IUI and IVF/ICSI
Developing simplified culture systems for clinical IVF
Implementing accessible infertility services into (existing) reproductive health care programmes.
The organization of training-courses
Continuous data registration
Advocacy and networking

## Objectives and approach

**Figure 4:**
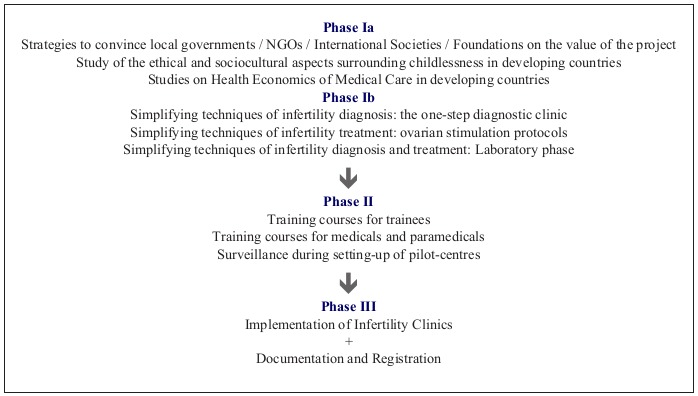
Overall strategy of the Walking Egg Project.

TWE npo aims to integrate infertility care within the concept of family planning, emphasizing that family planning is not only the prevention of unwanted pregnancies but to ‘safe proof’ a future chance of pregnancy in case of involuntary childlessness. The overall strategy of the Walking Egg project is summarized in Figure 4.

In the aftermath of the Arusha conference the Study Group Social Sciences further held expert meetings in 2009 and 2011 to discuss the sociocultural and ethical aspects of infertility care in DC. As a result of these meetings two Monographs were published in this journal (Ombelet and Van Balen, 2010; Gerrits et al., 2012).

Considering the overall strategy of TWE npo, up to now, phase Ia: strategies to convince governments, international societies, foundations and NGOs have been thus far insufficient and discouraging. Unsurprisingly, the main excuses against our goal and on-going work remain the misused arguments of ‘overpopulation’ and the ‘limited resources’, yet, not wondering where the resources needed to sustain growing populations in DC are being squandered (c.f. debate Matthew Connelly versus the United Nations director - https://thewalkingegg.com/video) (Ombelet, 2011).

In our strategy, we also carried out an internet-search for possible donors. For this, a questionnaire to learn about the actions and interest in infertility care in developing nations was sent to several important foundations, NGOs and international societies linked to reproductive health. Each and every organization responded with an overall interest in the issue of childlessness in DC, yet, in none of these organisations the issue of infertility care was being or planned to be addressed in the future. Likewise, considering local governments: it is not only the resource constraint which prevents to provide infertility services in many DC. As such, with the dominant discourse focusing on controlling overpopulation, or limited economical resources, it is no wonder that infertile women are marginalised and consequently excluded from health sector interventions (Ombelet, 2011).

In consequence, more initiatives to convince all stakeholders about the value of promoting reproductive health care, education and supporting the idea of universal access to infertility care are an important challenge currently and in the future.

**Figure 5:**
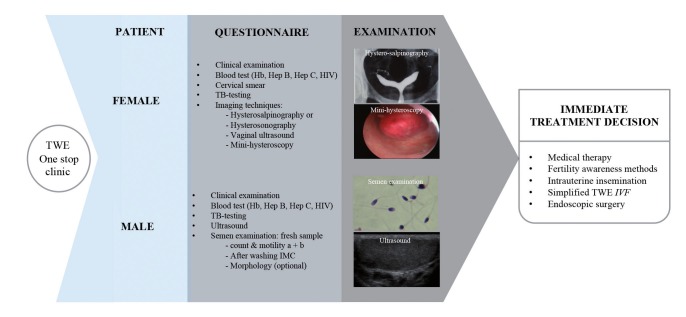
The Walking Egg One-stop diagnostic clinic.

In line with phase Ib, since 2007 many studies have been published supporting the value of one-stop diagnostic clinics, the effectiveness of low-cost mild ovarian stimulation protocols for in vitro fertilization (IVF) (Verberg et al., 2009; Ferraretti et al., 2015; Nargund et al., 2017), the value of intrauterine insemination (IUI) as a first step treatment option in selected cases (Cohlen et al., 2018) and the development of simplified IVF techniques (Van Blerkom et al., 2014). Regarding infertility diagnosis, the one stop clinic should be a cost-efficient structure providing accurate diagnostic services to a large demographic area. In one visit a complete evaluation of the fertility parameters are provided to determine a treatment strategy (Figure 5).

**Figure 6:**
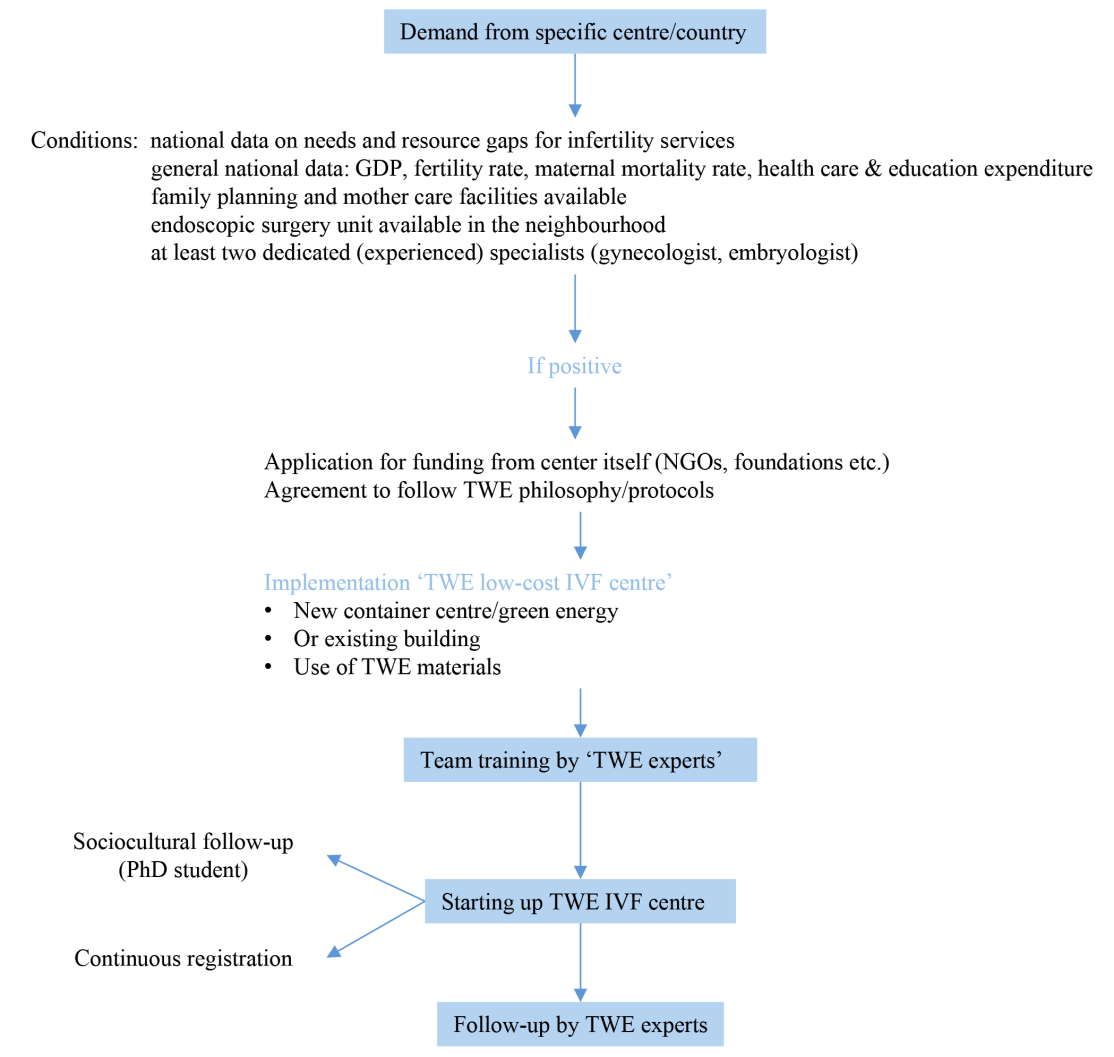
The different steps from application to implementation of Walking Egg Centres (TWE = The Walking Egg ; GDP = gross domestic product).

To provide treatment, as part of the Walking Egg Project and based on previous findings and experience, a new simplified method of IVF and embryo culture, called the TWE lab method has been developed, validated and become available for public and private IVF laboratories willing to include this treatment in their treatment offer (Van Blerkom et al., 2014). With this new system, the high costs of medical gases, complex incubation equipment and infrastructure, typical of IVF laboratories in high resource settings, can be avoided. Up to June 2019, a total of 180 babies have been born after using this technique with excellent and reassuring perinatal outcome results (results non-published). With this, the implementation of low-cost Walking Egg Centres is another major goal of our organization. Figure 6 gives an overview of the different steps from application to implementation of a Walking Egg Centre.

The implementation of a the Walking Egg Centre entails different activities and services including equipping the clinics, training the staff, administrative support, documentation, registration and follow-up of activities including psychological and socio-cultural follow-up of all patients. Table II gives an overview of the different key categories of the training courses.

**Table II:** — The main topics to be included in the training courses (Ombelet et al., 2013).

To study the ethical and sociocultural aspects of childlessness on a global scale	
	→ Target group: **nurses, midwifes**
A general and medical history of and basic clinical examination both partners	
	→ **clinician (medical)**
Screening for infections and STDs	
	→ **clinician (medical, paramedical)**
How to perform and evaluate a hysterosalpingography and/or hystero-salpingo-contrast-sonography	
	→ **clinician (medical, paramedical)**
Standard Operational Procedures for the gynaecological and fertility ultrasound scan	
	→ **clinician (medical, paramedical)**
Basic semenology training course according to WHO 2010 manual	
	→ **laboratory staff, (paramedical)**
Sperm washing procedures	
	→ **laboratory staff, (paramedical)**
Mini-hysteroscopy	
	→ **clinician (medical)**
Documentation and registration	
	→ **administrative staff (clerical)**

Considering the documentation and registration: strict follow-up and registration of all ART activities is mandatory within each pilot-centre by using on-line data. Administrative staff and (para) medicals have to be aware of the importance of correct and trustable data registration. The ultimate goal is to offer to all pilot-centres a customer-friendly registration programme. Hence, continuous monitoring of service activities will be centralized, and feed-back can be provided to the different centres for clinical and laboratory policy adjustments, information to couples on clinic performance, and information to society.

## Conclusion

Infertility treatment will likely become one of the most predominant components of future reproductive health care practices. As a matter of fact, as evidence-based, affordable solutions begin to drive global guidance within both public and private health care system solutions, access to care for the infertile couple will become one of the largest emerging fields in global medicine.

In this line, the Walking Egg npo has demonstrated in several studies and in the practice, the value of a one-day diagnostic clinic together with the safe low-cost simplified ovarian stimulation protocols and cost-effective simplified IVF laboratory systems to resolve infertility. We firmly believe that the worldwide implementation of such simplified methods, especially in resource-poor countries, will surely increase global access to infertility care.

Global access to infertility care is the key message of the Walking Egg Project. This objective can only succeed with the support of local policy makers as well as international societies. Furthermore, by taking advantage of current information and communication technologies (e.g. social media, open access scientific journals, etc...) to enhance accessibility and effectiveness of reproductive health care services and fertility awareness campaigns, ideally leading men and women in their reproductive years to ‘safe proof’ their fertility and/or to seek treatment timely, will also lead to the Walking Egg goal.

At present, as an estimation of the worldwide situation, the price to pay for IVF/ICSI, by patients themselves, the government or the insurance companies, varies between 4000 and 15000 € per cycle. If we can’t make these methods less expensive, IVF treatment will be (and is) limited to a small part of the world population (the happy few) creating a bigger cleavage and disparities between societies.

I cannot imagine that this was Bob Edwards’ dream when he offered IVF to the world.

**Figure:**
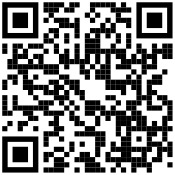


https://www.youtube.com/watch?v=QwYYMNn94Ws&feature=youtu.be
